# Pattern of Protein Expression in Developing Wheat Grains Identified through Proteomic Analysis

**DOI:** 10.3389/fpls.2017.00962

**Published:** 2017-06-09

**Authors:** Mingming Yang, Xiang Gao, Jian Dong, Nitant Gandhi, Huanjie Cai, Diter H. von Wettstein, Sachin Rustgi, Shanshan Wen

**Affiliations:** ^1^State Key Laboratory of Crop Stress Biology for Arid Areas, College of Agronomy, Northwest A&F UniversityYangling, China; ^2^Wheat Engineering Research Center of Shaanxi Province, Northwest A&F UniversityYangling, China; ^3^Department of Plant and Environmental Sciences, Clemson University Pee Dee Research and Education CenterFlorence, SC, United States; ^4^Key Laboratory of Agricultural Soil and Water Engineering in Arid and Semiarid Areas, Ministry of Education, Northwest A&F UniversityYangling, China; ^5^Institute of Water Saving Agriculture in Arid Regions of China, Northwest A&F UniversityYangling, China; ^6^Department of Crop and Soil Sciences, Washington State UniversityPullman, WA, United States

**Keywords:** wheat, grain development, metabolic pathway, proteome, chromosome

## Abstract

Grain development is one of the biological processes, which contributes to the final grain yield. To understand the molecular changes taking place during the early grain development, we profiled proteomes of two common wheat cultivars P271 and Chinese Spring (CS) with large and small grains, respectively at three grain developmental stages (4, 8, and 12 days post anthesis). An iTRAQ (isobaric tags for relative and absolute quantitation) based proteomics approach was used for this purpose. More than 3,600 proteins were reported to accumulate during early grain development in both wheat cultivars. Of these 3,600 proteins, 130 expressed differentially between two wheat cultivars, and 306 exhibited developmental stage-specific accumulation in either or both genotypes. Detailed bioinformatic analyses of differentially expressed proteins (DEPs) from the large- and small-grain wheat cultivars underscored the developmental differences observed between them and shed light on the molecular and cellular processes contributing to these differences. *In silico* localization of either or both sets of DEPs to wheat chromosomes exhibited a biased genomic distribution with chromosome 4D contributing largely to it. These results corresponded well with the earlier studies, performed in common wheat, where chromosome 4D was reported to harbor QTLs for yield contributing traits specifically grain length. Collectively, our results provide insight into the molecular processes taking place during early grain development, a knowledge, which may prove useful in improving wheat grain yield in the future.

## Introduction

Common wheat (*Triticum aestivum*, 2*n* = 6x = 42, genome AABBDD) is an allohexaploid with three subgenomes. It constitutes one of the major components of our daily diets and accounts for about 20% of calories consumed by the human beings (Brenchley et al., 2012; Pfeifer et al., 2014; Gu et al., 2015; Gao et al., 2016). Constantly increasing world population is imposing pressure on agriculture (Tester and Langridge, 2010), which makes it imperative to improve our understanding of the basic plant processes, such as grain development that directly or indirectly contributes to the final grain yield. It is relatively difficult to decipher the molecular biology of grain development in common wheat due to the sheer size and complexity of its genome with 104,091 identified protein-coding genes (Clavijo et al., 2017).

Grain development in wheat is a complex process, which can be partitioned into three consecutive phases: cellularization, effective grain-filling, and maturation (Nadaud et al., 2010). The first stage largely includes cellularization and differentiation (Sabelli and Larkins, 2009). It is an established fact that 80% of the final grain size is determined during this phase by the number of endosperm cell divisions and proliferation of the resulting cells (Brocklehurst, 1977; Laudencia-Chingcuanco et al., 2007; Sabelli and Larkins, 2009; Nadaud et al., 2010). During grain development a major transition point occurs at about 12 days post anthesis, essentially it marks the end of endosperm cell division or the first phase (Mechin et al., 2007; Shewry et al., 2012) and start of the grain filling or second phase. During this phase accumulation of storage compounds like starch and gluten proteins take place. A complex gene network regulates protein expression during grain filling (Gutierrez et al., 2007; Nadaud et al., 2010). Many transcriptomics, proteomics, and metabolomics approaches have been so far used to understand the biochemical processes taking place during the early grain development in common wheat.

Proteomics is a powerful tool to understand the molecular processes taking place during grain development. In the last decade proteomic approaches have been extensively used in common wheat and barley to investigate the grain development under well watered and drought prone conditions (Dupont, 2008; Dupont et al., 2011; Ge et al., 2012; Guo et al., 2012; Kaspar-Schoenefeld et al., 2016; Yang et al., 2016). Among various proteomics approaches isobaric tag for relative and absolute quantitation (iTRAQ) of proteins permits reliable qualitative and quantitative profiling of many more proteins, as was earlier possible. Moreover, with sufficient number of proteins identified through this approach it is now possible to conduct detailed pathway analysis and investigate protein-protein interactions (Thingholm et al., 2009; Wang and You, 2012; Zi et al., 2013).

The former research showed that seed development in Arabidopsis and canola shares a similar pattern, which involves an early proliferation of endosperm to form a large seed cavity (i.e., embryo sac), followed by a second phase of development in which the embryo grows to replace the endosperm (Xiao et al., 2016). A significant body of research on grain development has been also conducted in cereals, specifically in wheat, albeit the understanding of molecular processes taking place during early grain development is still far from complete. In this study, we performed the first integrated comparative proteome analysis by iTRAQ during the early wheat grain development under the field conditions using two wheat cultivars with contrasting grain characteristics. A considerable number of proteins were identified to show differences in their relative abundances in two wheat cultivars. These results provide new insights into the central metabolic changes taking place during early grain development in common wheat. Moreover, this work demonstrated that wheat grain undergoes significant molecular changes over the examined period of time and provides useful information, which can be implemented in fostering the future breeding activities focused on the development of high yielding wheat cultivars.

## Materials and methods

### Plant material and growth conditions

Wheat cultivars P271 and Chinese Spring (CS) were grown at the Yangling Experimental Station located at 34.26°N, 108.14°E in the Shaanxi Province during 2013–2014 wheat growing season. Plants were fertilized with urea (60 Kg/ha) and watered periodically. The main culm spikes were tagged upon anthesis, and the labeled spikes were sampled at 4, 8, and 12 days post anthesis (DPA). Grains were sampled from the four spikelets at the center of each spike. Samples from each stage consisted of at least 200 seeds from 30 spikes. Samples were snap frozen in liquid nitrogen and stored at −80°C for later use (Li et al., 2012). Two biological and three technical replicates were analyzed at each developmental time point to minimize experimental errors and number of false positives.

### Measurement of physiological parameters

Grains were collected at different development stages (4, 8, and 12 DPA) from P271 and CS, and lengths of 10 grains in three replicates were recorded. Data was subjected to statistical analysis using Statistix 10.0 package.

### Morphological and cytological characterization of the wheat grains

Wheat grain size measurements were taken as described in Disch et al. (2006). Briefly, seeds of P271 and CS at different developmental stages were collected, in freshly prepared FAA [10% formalin (v/v), 5% acetic acid (v/v), and 45% ethanol (v/v)] and incubated at 4°C for 2 h. After incubation samples were washed for five times with 0.1% PBS (Phosphate buffer saline), and fixed in osmic acid at 4°C for 2 h. Following this step samples were washed again for five times with 0.1% PBS. Subsequently samples were dehydrated by putting in a series of alcohol dilutions: 30, 50, 75, 95, and 100% for 30 min each, and finally immersed in acetone for 1 h. Following dehydration the samples were embedded in various proportions of Epon812 and acetone: 1:3 for 3 h, 1:1 for 4 h, and 3:1 over night. After this step samples were dried at 30°C for 24 h and 60°C over night. Subsequently the embedded tissue was sectioned into 1–2 μm slices by Leica microtome, transferred to slide and mounted in distilled water. Later the slides were incubated at 60–80°C for drying. Subsequently 1% toluidine blue solution pH 5.5 was added. Following this step slides were analyzed under the microscope and pictured with the digital camera (Olympus, System Microscope BX53) following Ma et al. (2015).

### Protein extraction

Frozen wheat grains were pulverized in liquid nitrogen. After pulverization samples were suspended in 10 ml ice-cold phenol extraction buffer [0.7 M sucrose; 0.1 M KCl; 50 mM EDTA, 0.5 M Tris-HCl, 1% (w/v) DTT, 0.1 mM PMSF, pH 7.5], and incubated at 4°C for 30 min. During incubation the mixture was vortexed for 10 s every 5 min. After incubation the mixture was centrifuged at 5,000 × g for 30 min under refrigeration. Following centrifugation the phenolic phase was collected and precipitated overnight with five volumes of 100 mM ammonium acetate prepared in methanol at −20°C. After precipitation the mixture was centrifuged at 5,000 × g for 30 min at 4°C, and the supernatant was discarded. The resultant pellet was rinsed twice with ice-cold acetone with 0.2% DTT (w/v). Following this step the pellet was air-dried and resuspended in 200 μl of RIPA lysis buffer [50 mM Tris-HCl (pH 8.0), 150 mM NaCl, 1% SDS, and 0.1% Triton 100]. Subsequently the protein concentration was determined by standard BCA (bicinchoninic acid) assay using bovine serum albumin as standard (Beyotime Company, Shanghai). Following extraction, the protein solution was incubated at 60°C for 1 h in presence of 2% DTT (w/v). After reduction the free cysteines were alkylated in the presence of 7.5 mM iodoacetamide by incubating the mixture at room temperature for 10 min in the dark.

### iTRAQ labeling

One hundred microgram protein from each sample was digested overnight with trypsin (Promega) at 37°C. Following digestion the protein samples were labeled with 8-plex iTRAQ kit (Applied Biosystems). Briefly, one unit of iTRAQ reagent (defined as the amount of reagent required to label 100 μg of protein) was thawed and reconstituted in 70 μl isopropanol. Peptides from different treatments were labeled with different iTRAQ tags, respectively, by incubation at room temperature for 2 h (see Table S1 for details). The labeled peptide were then pooled and dried by vacuum centrifugation. The pooled mixture of iTRAQ-labeled peptides was fractionated by high-pH reversed-phase chromatography.

### High-pH reversed-phase chromatography

Isobaric tag for relative and absolute quantitation (iTRAQ) labeled samples were resuspended in 100 μl of 20 mM HCOONH_4_ (ammonium formate) with 2 M NaOH (pH 10), and separated on a Dionex UltiMate 3,000 RSLCnano system (Thermo Fisher Scientific) equipped with a Gemini® 3 μm NX-C18, 110 Å, 75 × 2 mm LC Column (Phenomenex). Peptides were resolved on a linear gradient of 5–40% of mobile phase B [20 mM HCOONH_4_, 2 M NaOH and 80% acetonitrile (ACN)] in mobile phase A (20 mM HCOONH_4_ and 2 M NaOH) in a duration of 30 min at a flow rate of 0.2 ml/min. The UV detector was set at 214/280 nm, and fractions were collected every 1 min. In total, 24 fractions were collected and dried by vacuum dryer.

### Electrospray ionization (ESI) tandem MS (MS/MS) analysis by Q exactive

After acidification with 50% CF_3_COOH (trifluoroacetic acid, TFA), a linear gradient of 5% to 90% of mobile phase B [80% ACN and 0.1% formic acid (FA)] in mobile phase A (5% ACN and 0.1% FA) at a flow rate of 300 μl/min for 50 min was used to separate the peptides followed by their ionization. Subsequently, the MS/MS analysis was performed on a Q Exactive system (Thermo Scientic) in the Information Dependent Mode. MS spectra were acquired across the mass range of 350–1800 m/z in the high-resolution mode (>70,000) and the 40 ms accumulation time per spectrum. A maximum of 20 precursors per cycle were chosen for fragmentation from each MS spectrum with 60 ms minimum accumulation time for each precursor and dynamic exclusion for 20 s. Tandem mass spectra were recorded in the high sensitivity mode (resolution >17,500) with rolling collision energy on and iTRAQ reagent collision energy adjustment on.

### Sequence database searching and data analysis

To identify the proteins, the MS/MS spectra were processed by ProteinPilot™ Software 4.5 (AB Sciex). Following criteria were upended for protein identification and quantification: MS tolerance value of 0.05 Dalton (standard deviation 0.0025 Dalton), MS/MS tolerance value of 0.10 Dalton (standard deviation 0.004 Dalton), two unique peptides identified in at least two of the three technical replicates, cysteine carbamidomethylation as fixed modification, and methionine oxidation as variable modification. The acquired MS/MS spectra were automatically searched against a total of 75,015 wheat protein sequences (Wheat_2015.4.17_UN.fasta) available in the UniProt reference database (http://www.uniprot.org/proteomes/UP000019116). Protein quantification was based on the abundances of reporter tags, which reflect the relative ratio of the peptide in the samples that were combined. In addition, the following criteria were set forth to identify differentially expressed proteins (DEPs): (i) all identified proteins must exhibit ≥95% confidence on the protein confidence threshold cutoff of 1.3 (unused), and (ii) fold-change of >2 or <0.5 were set as cutoff value to call a change in protein abundance significant.

### Bioinformatics

The differentially expressed proteins were examined using AgriGO for gene ontology (GO) annotation and enrichment analyses. The GO project classifies proteins in three categories based on their annotation: biological process, cellular component and molecular function. Following annotation, the enzyme codes assigned to each protein were sequentially mapped to the known metabolic pathways in the Kyoto Encyclopedia of Genes and Genomes (KEGG, http://www.genome.jp/kegg/) (Kanehisa et al., 2012).

### Genomic distribution of DEPs

Genomic distribution of DEPs was studied by assigning them to wheat sub-genomes, homoeologous chromosome groups and specific chromosomes. For this purpose, Sequences of DEPs were blasted against the wheat genomic DNA sequences available in the public domain. To eliminate the bias of genome size or gene number (predicted per chromosome) an expected value of DEPs was calculated for each chromosome using the following formula: (total number of genes or genome size/total number of DEPs) × total number of DEPs predicted per chromosome. Expected and observed numbers of DEPs per chromosome were plotted in the form of bar diagrams, and the two bar diagrams (one based on the gene number and the other based on the genome size) were compared.

## Results

### Morphological and cytological differences in the early grain development of wheat cultivars P271 and chinese spring (CS)

**Figure 1:**
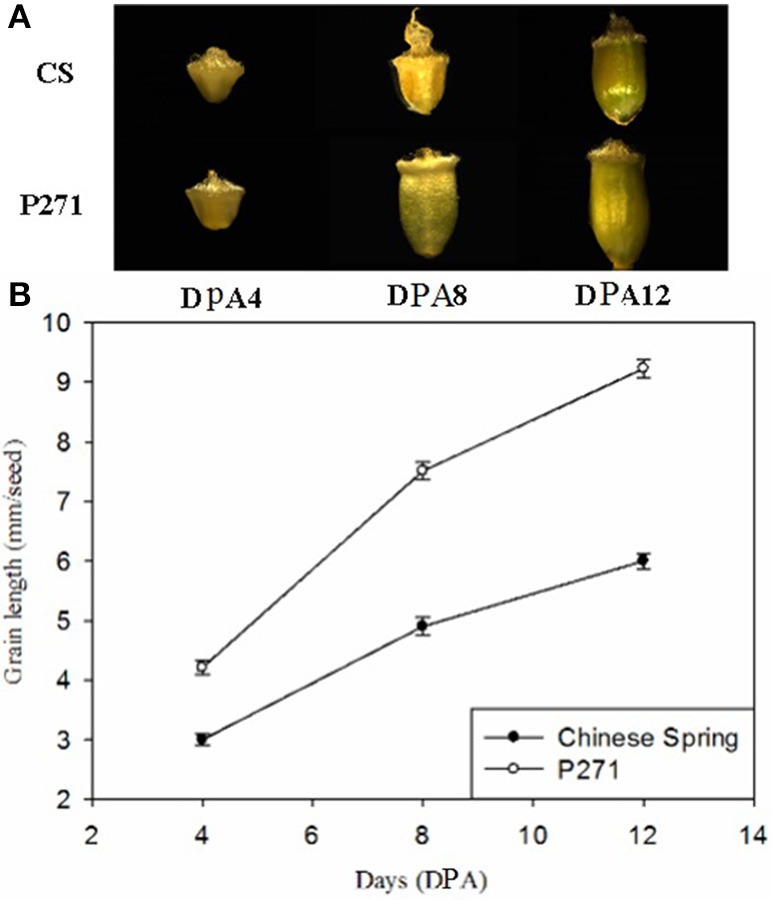
Phases of grain development in wheat cultivars P271 and Chinese Spring (CS). **(A)** Grain development between 4 days post anthesis (DPA) to 12 DPA. **(B)** Changes in grain length between 4 and 12 DPA.

In general, grain size and weight in both P271 and CS increased significantly from 4 to 12 DPA. But the rate of grain development differed between the two cultivars. P271 exhibited a larger grain size than CS from 4 to 12 DPA, which corresponded well with its final grain size (Figure 1).

Transverse sections of developing caryopses of wheat cultivars P271 and CS at 4, 8, and 12 DPA were observed under microscope and pictured. Lower panels in the Figures S1A,B showed complete transverse sections of developing grains at 4, 8, and 12 DPA, and the upper panels, respectively showed higher magnification images of areas in the peripheral endosperm. It is apparent from looking at the transections of the immature grains that the shape and size of ovary and ovule are different in the two cultivars (Figures S1A,B), which largely dictate endosperm shape and size. In CS, at the 8 DPA, endosperm cellularized to near completion and enlarged significantly in size giving it a sickle shape. In this process endosperm crushed the integuments and nucellus and filled the space inside the pericarp (ovary walls) (Figures S1A). On the other hand, in P271, at 8 DPA, the endosperm that exhibited a kidney shape stayed partly cellularized and unexpended leaving a cavity between seed (ovule) and pericarp (see Figure S1B). In P271 enlargement of endosperm took place between 8 and 12 DPA, and at this stage it occupied all space between seed and pericarp filling up the cavity. These embryological differences or phenotypic markers underpin the developmental differences between the two common wheat cultivars with contrasting grain characteristics.

### Differences in the protein profiles of P271 and chinese spring (CS) during early grain development

In this study, iTRAQ-based quantitative proteome characterization approach was used to investigate early grain developmental differences in two wheat cultivars P271 and CS with contrasting grain characteristics. For this purpose protein profiles at three development stages 4, 8, and 12 DPA were obtained. A total of 3,690 proteins were identified in CS, and 3,680 proteins were identified in P271. As shown in Figure S2, 3,678 proteins were detected in both wheat cultivars, whereas 12 proteins in CS and 2 proteins in P271 accumulated exclusively in two wheat cultivars (cf. Table S2). Of these 12 CS specific proteins 7 showed up-regulation and 5 showed down-regulation. More specifically, a ferredoxin thioredoxin reductase (W5GA08) showed 4.2-fold up-regulation during early grain development in CS. Both of the proteins that uniquely accumulated in P271 exhibited up-regulation (Table S2).

**Figure 2:**
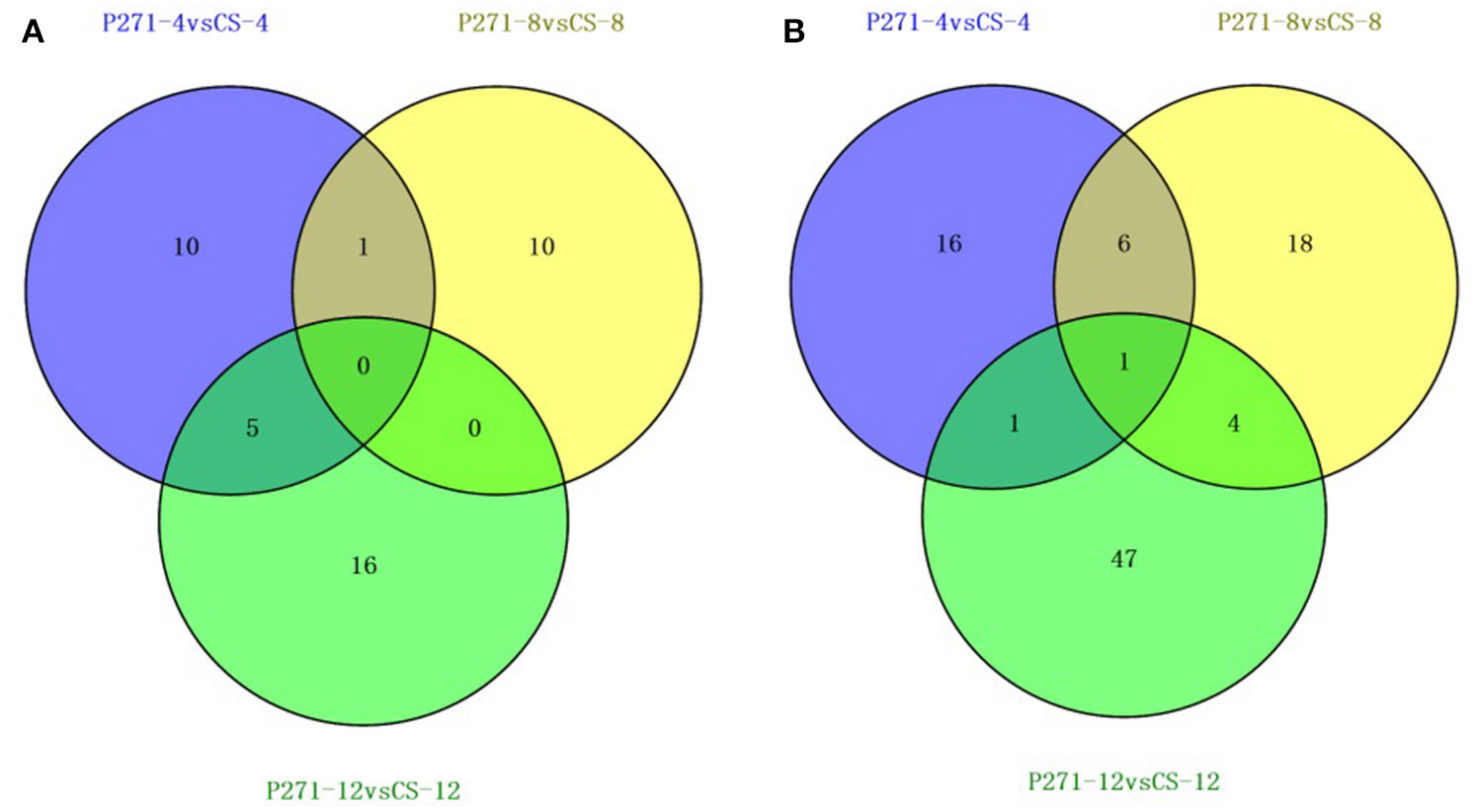
Venn diagrams showing number of differentially expressed proteins identified from developing grains of wheat cultivars P271 and CS at 4, 8, and 12 days post anthesis (DPA): Proteins showing up-regulation **(A)**, and down-regulation **(B)**.

A 2-fold cut-off was used to implicate significant changes in the abundance of a protein and to classify it as a differentially expressed protein (DEP). In one to one comparisons between CS and P271, a total of 130 proteins showed more than 2-fold change (*p* ≤ 0.05) in their respective abundances at least at one of the three studied time points in the early grain development (Tables S3–S5). Distribution of 130 DEPs with overlaps among three developmental stages was illustrated in Figure 2. Of 130 DEPs detected in the CS-P271 comparison, 91 DEPs exhibited up regulation (Figure 2A) and 42 DEPs showed down regulation (Figure 2B) at least at one of the three studied time points in the early grain development.

### Differential accumulation of proteins at different grain developmental stages in P271 and chinese spring (CS)

**Figure 3:**
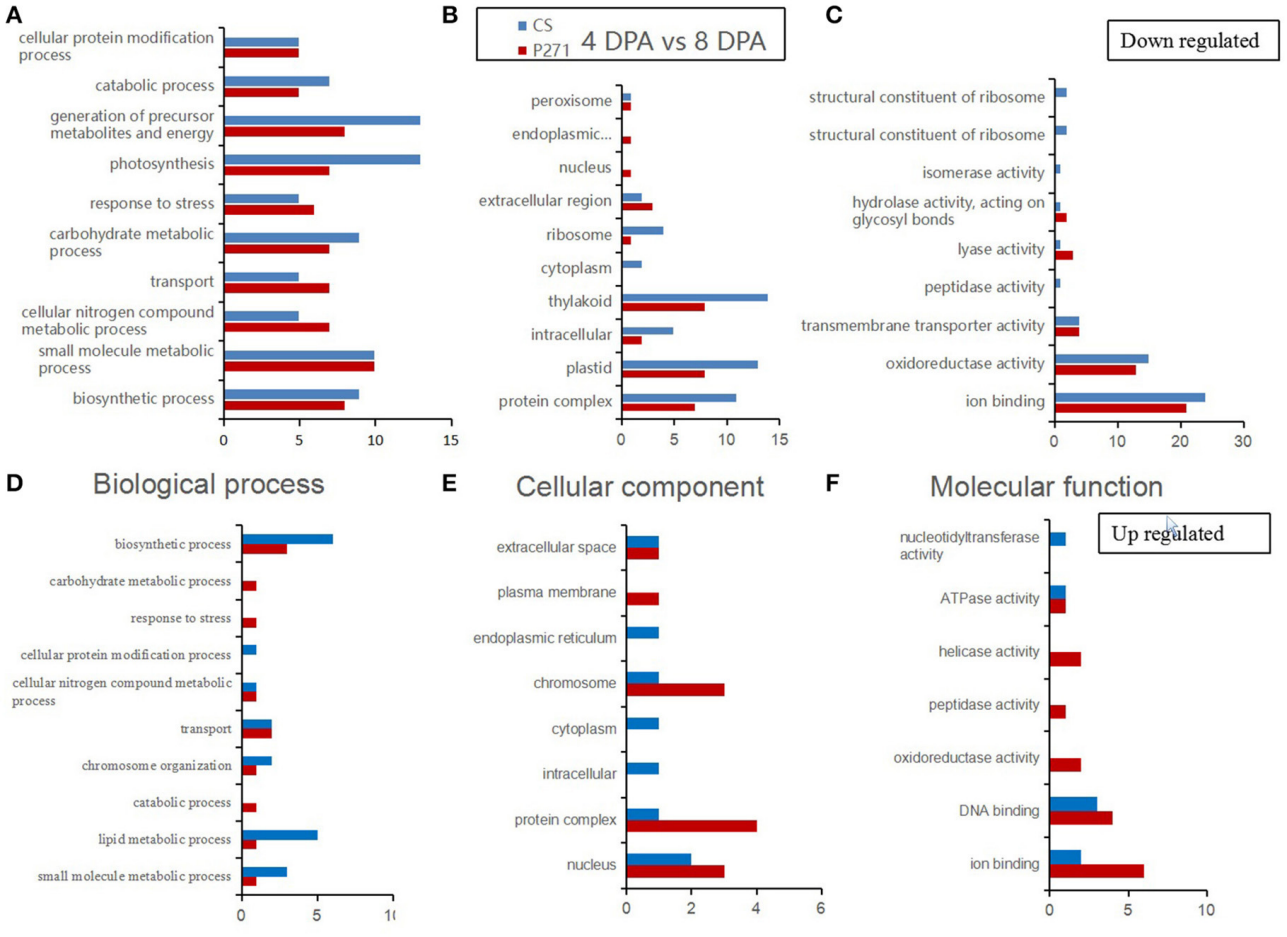
Bar diagrams showing distribution of differentially expressed proteins in wheat cultivars CS and P271 in the 4 vs. 8 DPA (days post anthesis) comparison. The proteins were classified based on their predicted functions into: Biological process, **(A)** down regulated and **(D)** up regulated; Cellular component, **(B)** down regulated and **(E)** up regulated; and Molecular function, **(C)** down regulated and **(F)** up regulated.

**Figure 4:**
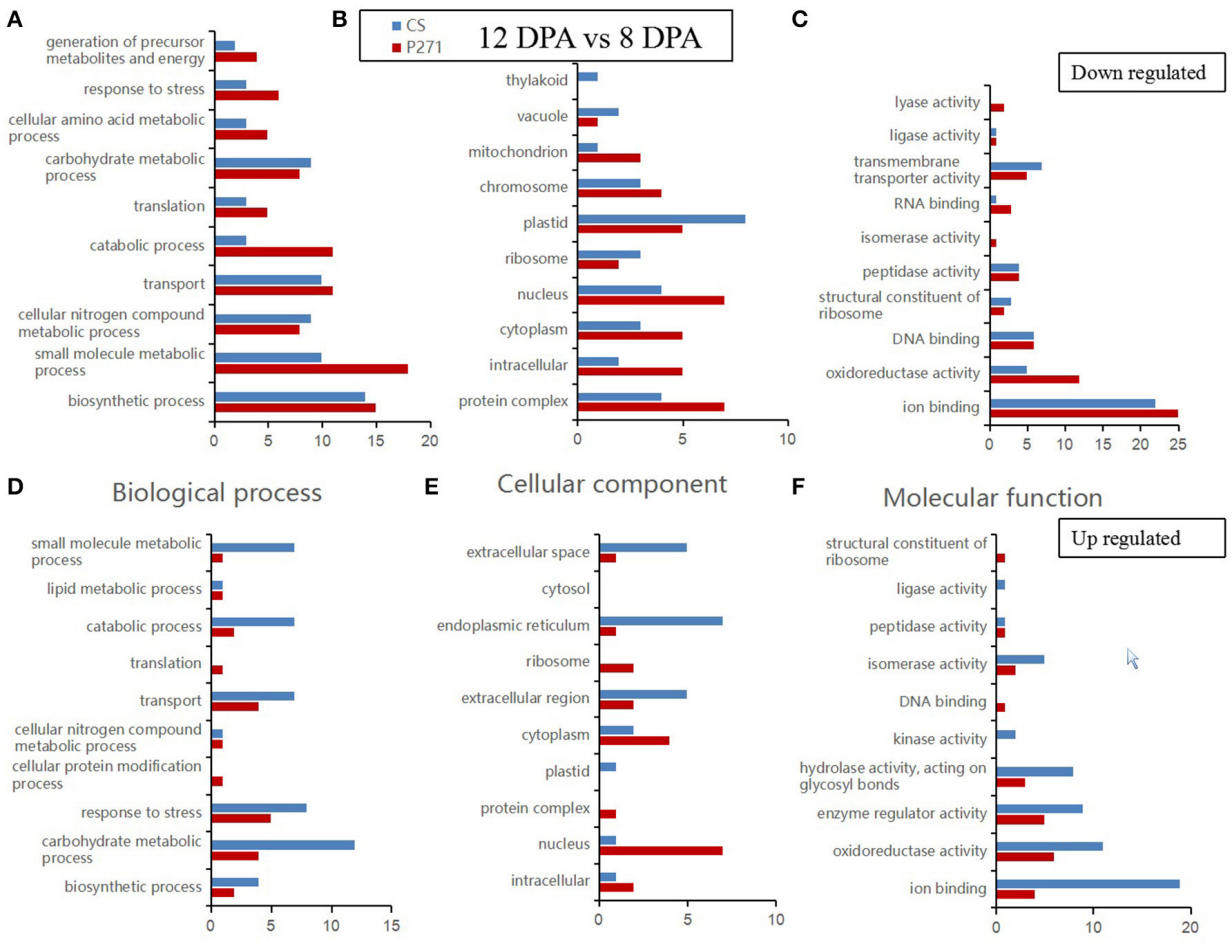
Bar diagrams showing distribution of differentially expressed proteins in wheat cultivars CS and P271 in the 12 vs. 8 DPA (days post anthesis) comparison. The proteins were classified based on their predicted functions into: Biological process, **(A)** down regulated and **(D)** up regulated; Cellular component, **(B)** down regulated and **(E)** up regulated; and Molecular function, **(C)** down regulated and **(F)** up regulated.

A GO category enrichment analysis was performed on DEPs detected in one to one comparisons between two-grain developmental stages: 4 and 8 DPA or 12 and 8 DPA in CS and P271. The purpose of this analysis was to determine the overall trend of enrichment in the specific functional categories during the early grain development in two wheat cultivars. In this analysis DEPs were categorized according to the GO Slim classification for plants. The GO annotations of DEPs in the three functional categories namely “biological process,” “cellular component,” and “molecular function” from 4 vs. 8 DPA to 8 vs. 12 DPA comparisons are, respectively shown in Figures 3, 4.

In the first comparison, i.e., 4 vs. 8 DPA, most proteins were found to classify in the category “biological processes.” When compared with P271, in CS, more up regulated DEPs were found to fall under subcategories “small molecule metabolic process,” “lipid metabolic process” and “biosynthetic process,” and more down-regulated DEPs in subcategories “photosynthesis,” “catabolic process,” “carbohydrate metabolic process,” and “generation of precursor metabolites and energy.” In the cellular component category, more DEPs were found to be down regulated than up regulated in the 4 vs. 8 DPA comparison in both wheat cultivars. Compared to P271, in CS, more down-regulated DEPs fall under the subcategories “thylakoid,” “plastid,”and “protein complex.” On the other hand, when compared to CS, in P271, more up regulated DEPs were found to fall under subcategories “protein complex,” “nucleus,” “plasma membrane,” and “chromosome.” In molecular function category, more DEPs were found to be down regulated than the up regulated in both wheat cultivars. However, more proteins from P271 than CS marked their presence in up-regulated DEPs. Collectively, in both cultivars, more DEPs were found to fall under the subcategories “ion binding” and “oxidoreductase activity.”

In the second comparison, i.e., 12 vs. 8 DPA, under the biological process category, more DEPs involved in “carbohydrate metabolic process” showed up regulation in CS, and almost similar number of DEPs involved in “biosynthetic process” showed down-regulation in both cultivars. In the cellular component category, in comparison with CS more DEPs in P271 belonging to subcategories “protein complex,” “intracellular,” “cytoplasm,” “nucleus,” “mitochondrion,” and “chromosome” exhibited down regulation. Interestingly, more DEPs belonging to some of above mentioned subcategories, such as “intracellular,” “nucleus,” “cytoplasm,” and “protein complex” also showed up regulation in P271. In contrast more up-regulated DEPs in CS fall under the subcategory “extracellular space,” “endoplasmic reticulum,” and “extracellular region.” Similar to the first comparison (4 vs. 8 DPA), most DEPs in the second comparison (12 vs. 8 DPA) were also found to be involved in “ion binding” and “oxidoreductase activity” subcategories under the “Molecular function” category. Collectively, in both cultivars, more up regulated DEPs were found to classify in subcategories “enzyme regulator activity,” “hydrolase activity, acting on glycosyl bonds” and “isomerase activity,” and down regulated DEPs in subcategories “DNA binding,” “peptidase activity,” “transmembrane transporter activity,” and “structural constituent of ribosome.”

**Figure 5:**
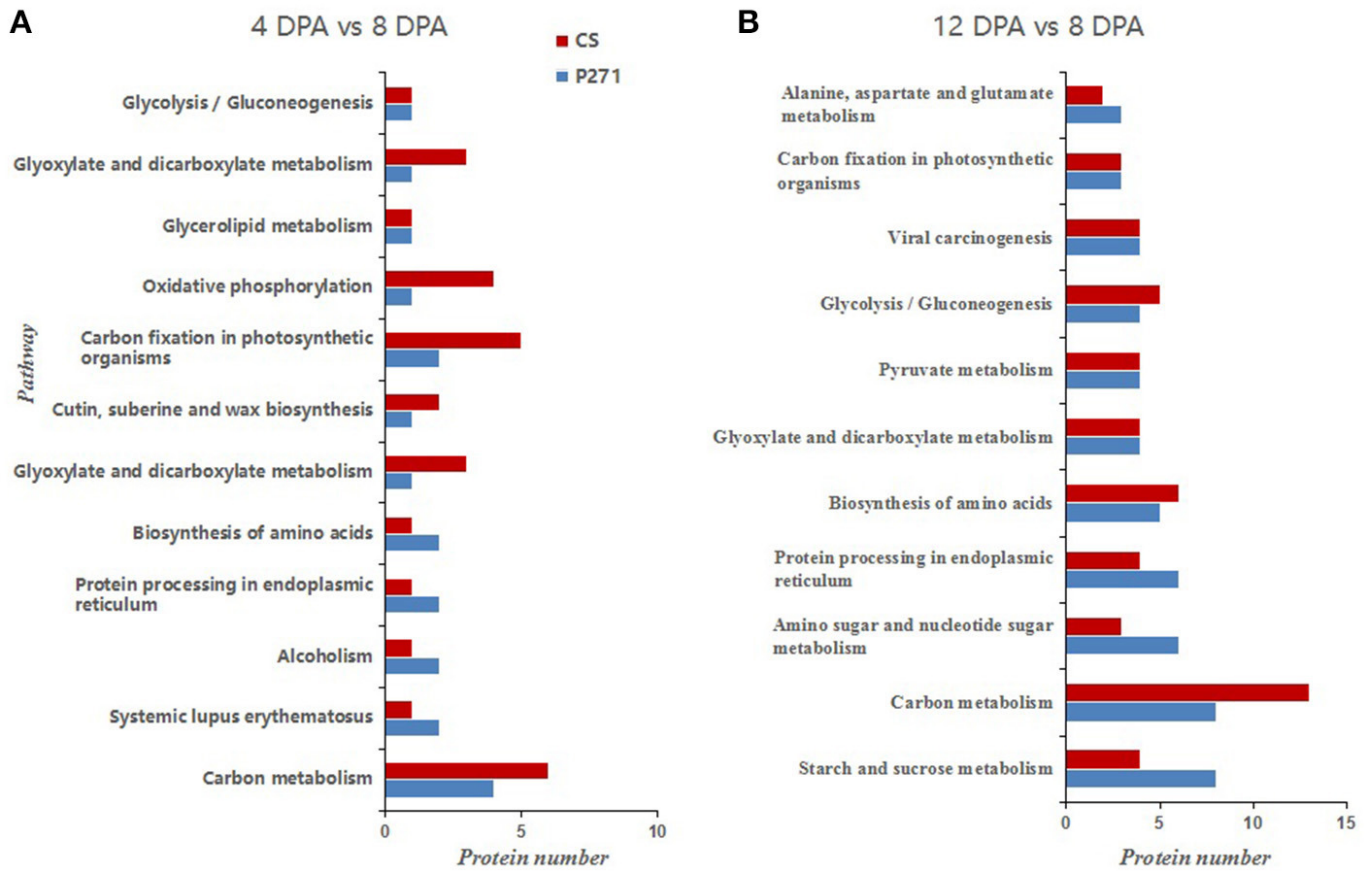
Bar diagrams showing distribution of differentially expressed proteins involved in pathways by KEGG analysis in P271 and CS. **(A)** DPA 4 vs. DPA 8 in CS and P271, **(B)** DPA 12 vs. DPA 8 in CS and P271.

It is apparent from the results of KEGG analysis that DEPs mainly belong to the following categories: carbon metabolism, amino acid biosynthesis, protein processing in endoplasmic reticulum and carbon fixation in photosynthetic organisms (Figure 5). When compared with CS, more proteins were found to fall under the following categories in P271 in 12 vs. 8 DPA comparison: starch and sucrose metabolism, amino sugar and nucleotide sugar metabolism and protein processing in endoplasmic reticulum. This observation might have some implications in the determination of the final grain size in P271. Similarly, in 12 vs. 8 DPA comparison, more carbon metabolism related proteins were found to accumulate in CS. A similar trend was also observed in P271 albeit at an earlier developmental stage (in 4 vs. 8 DPA comparison).

### Protein co-expression analyses during early grain development of P271 and chinese spring (CS)

**Figure 6:**
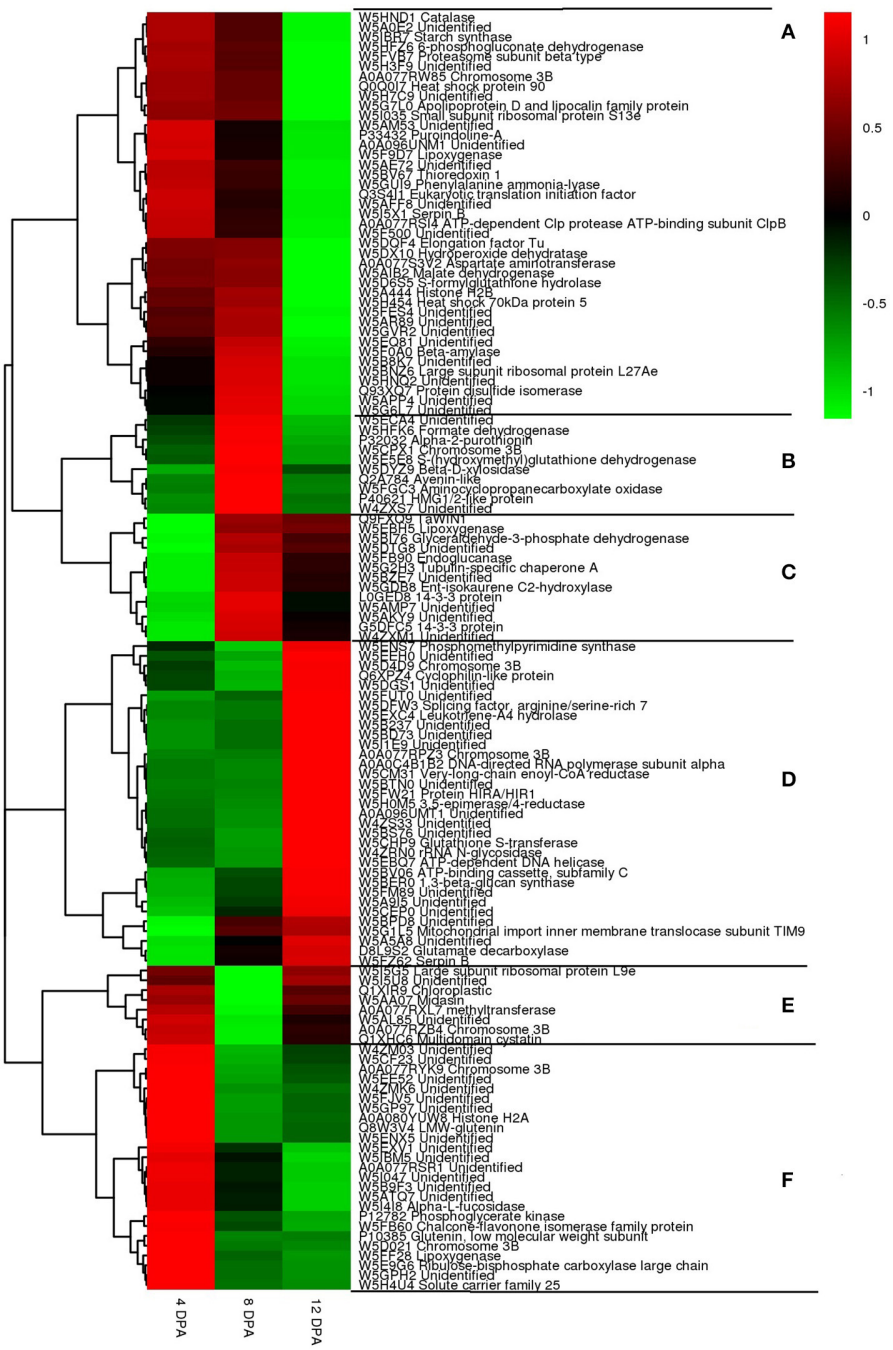
Hirerarchical clustering of 143 differentially expressed proteins (DEPs) at three grain developmental stages in wheat cultivars P271 and CS. **(A)** down-regulation; **(B)** down-up-down regulation; **(C)** down-up regulation; **(D)** down-down-up regulation; **(E)** up-down-up regulation; **(F)** up-down-down regulaiton. Red = up-regulated and green = down-regulated.

Expression profiles of 130 DEPs, which co-accumulated in the two wheat cultivars at the three developmental stages: 4, 8, and 12 DPA were investigated by hierarchical cluster analysis (Figure 6). Five major expression patterns (A–F) were observed that clearly exhibited differences among different developmental stages.

Expression pattern “A” included 41 proteins that exhibited a trend of down-regulation. This cluster was consisted of many metabolic enzymes, heat-shock proteins and plant defense proteins. Expression pattern “B” included 10 proteins that exhibited a trend of down-up-down regulation. In this cluster most of the proteins were related to metabolic enzymes or storage proteins. Expression pattern “C” showed a trend of down to up regulation. This cluster was comprised of 13 proteins, which mainly included enzymes involved in glyceraldehyde-3-phosphate dehydrogenase (GAPDH), tubulin-specific chaperone A and 14-3-3 protein. Expression pattern “D” showed a trend of down-down-up regulation. This cluster was consisted of 33 proteins, which includes phosphomethylpyrimidine synthase, cyclophilin-like protein, glutathione S-transferase and 1,3-beta-glucan synthase. Expression pattern “E” showed up-down-up regulation. The cluster included 8 proteins mainly related to large subunit ribosomal protein, midasin and methltransferase. Expression pattern “F” showed up-down-down regulation. The cluster included 25 proteins mainly related to low molecular weight glutenins, alpha-L-fucosidase, chalcone-flavonone isomerase family proteins, etc.

### Genomic distribution of DEPs

**Figure 7:**
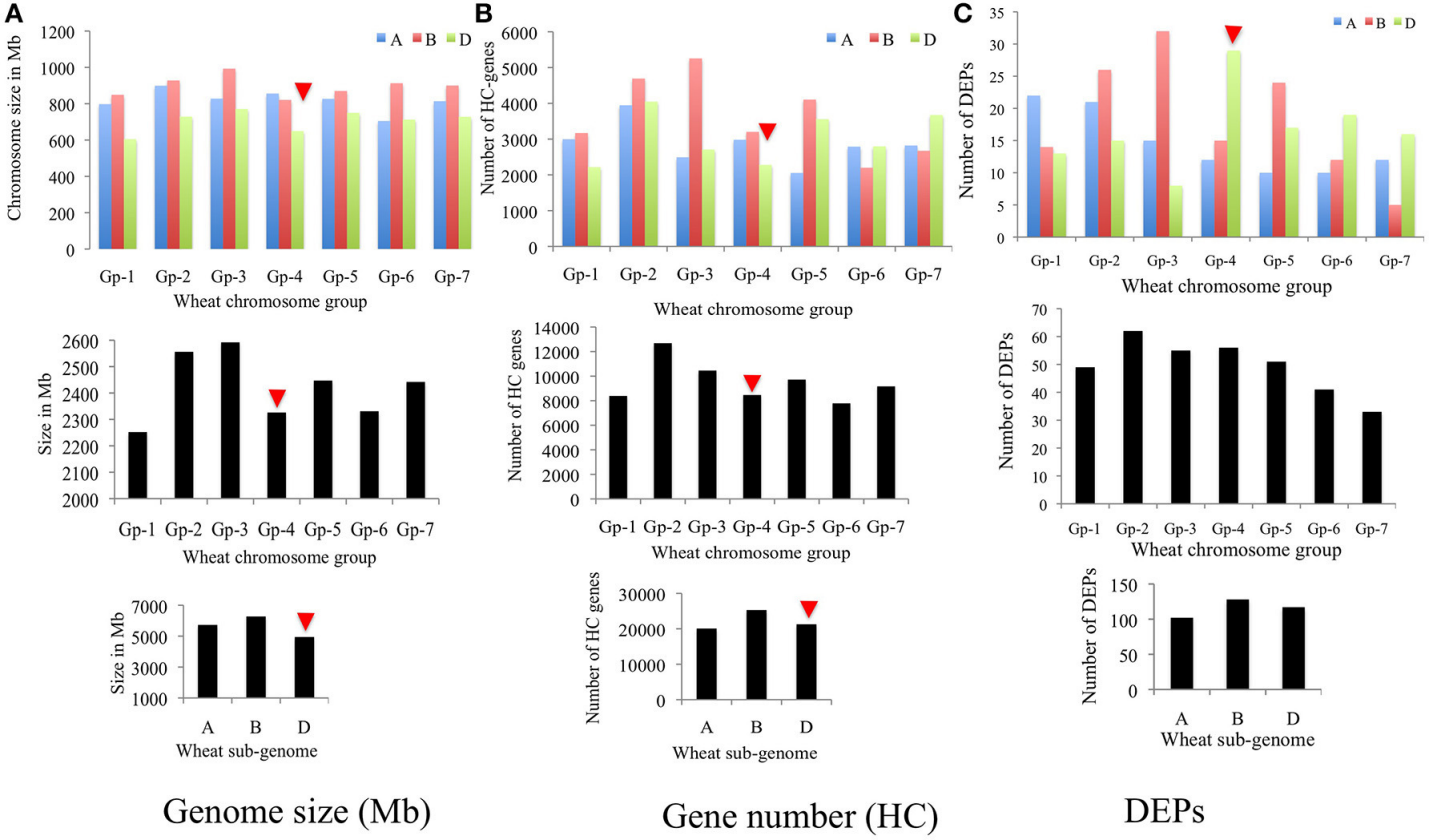
Bar diagrams showing genomic distribution of differentially expressed proteins (total 347) in wheat: **(A)** subgenomes, **(B)** homoeologous chromosome groups, and **(C)** specific chromosomes.

Genomic distribution of 347 DEPs [i.e., (306 DEPs + 130 DEPs)–89 overlapping DEPs; cf. Figure S3] was studied by assigning them to specific sub-genomes, homoeologous chromosome groups and specific chromosomes (Figures 7A–C). Results of this analyses unveiled that B subgenome of common wheat encoded more DEPs (128) than D (117) and A (102) subgenomes. An observation that corresponded well with the high predicted gene number (25,298–high confidence or HC genes) and large genome size (6,274 Mb) of B subgenome. In contrary, the D sub-genome, which is smallest in size (4,945 Mb) and carries almost equal number (21,277) of genes to the A subgenome (20,078) encoded significantly more DEPs (117) than the A subgenome (102). When distribution of DEPs in homoeologous chromosome groups was studied group 2 and group 4 chromosomes exhibited a biased distribution.

**Figure 8:**
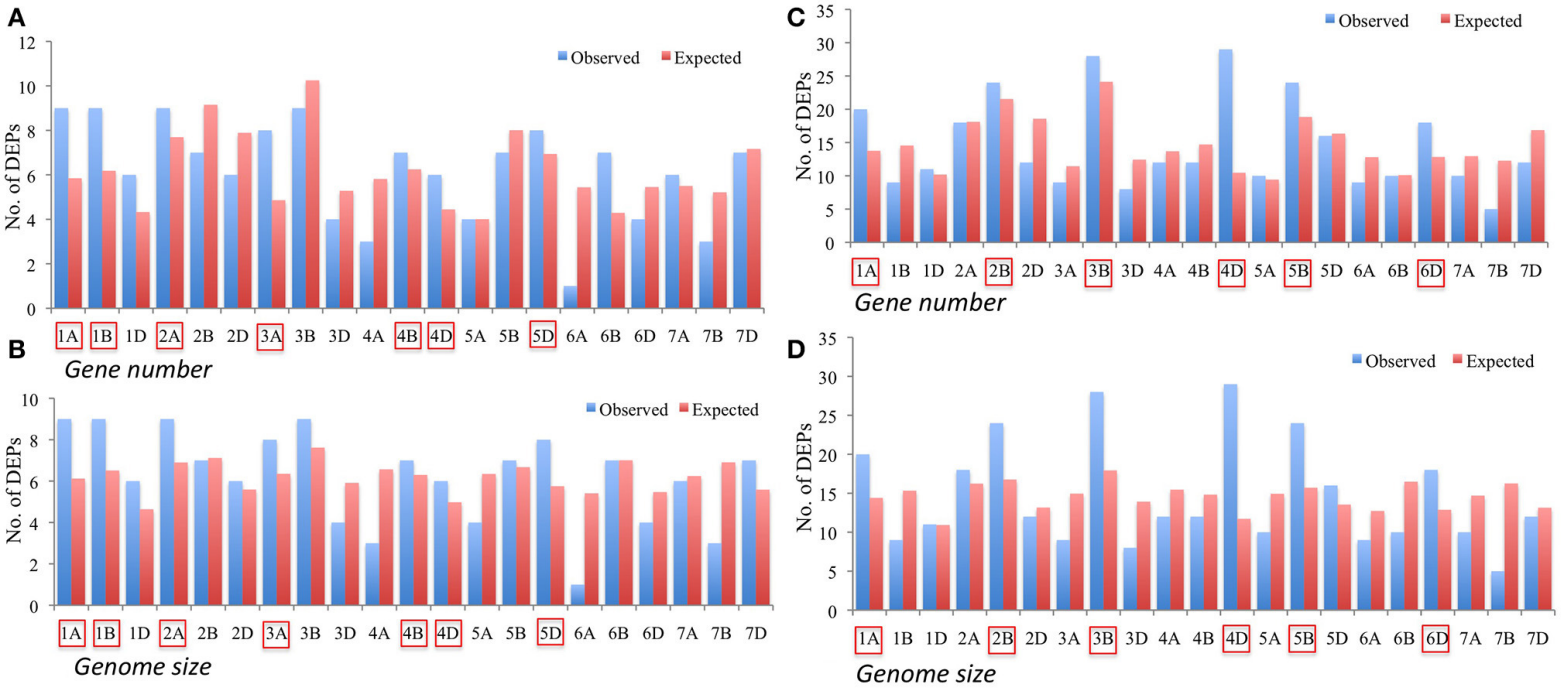
Bar diagrams showing distribution of differentially expressed proteins (DEPs) on specific chromosomes with expected number of DEPs calculated based on genome size **(A,C)** and gene number **(B,D)**. Panels **(A,B)** represent distribution of 130 DEPs identified from a comparison between CS and P271, whereas Panels **(C,D)** show distribution of 306 DEPs identified from the comparisons made between different developmental stages (4 vs. 8 DPA and 12 vs. 8 DPA). Chromosomes exhibiting more observed than expected number of DEPs are marked.

However, to get the better understanding of the genomic distribution of genes contributing to DEPs, the two sets of DEPs i.e., 130 DEPs identified from the CS vs. P271 comparison and 306 DEPs identified from the developmental stage specific comparisons (4 vs. 8 DPA and 12 vs. 8 DPA) were separately assigned to wheat chromosomes. In the former set of DEPs, more proteins were found to be contributed by the group 1 and group 2 chromosomes (Figure S4), and in the later set by group 2 and group 4 chromosomes (Figure S5). In each set of DEPs, the expected and the observed numbers of DEPs per chromosome were plotted in the form of a bar diagram, and the two bar diagrams thus obtained were compared (Figures 8A–D). In the former set of DEPs, chromosomes 1A, 1B, 2A, 3A, 4B, 4D, and 5D showed more observed vs. expected number of DEPs (Figures 8A,B). On the other hand, in the later set of DEPs, chromosomes 1A, 2B, 3B, 4D, 5B, and 6D exhibited more observed vs. expected DEPs. Among these chromosomes 4D exhibited a greater bias in both sets of DEPs.

In order to understand the dynamics of expression of DEPs over the studied period of time, the two sets of DEPs were assigned to specific chromosomes and the number of DEPs mapping to each chromosome was plotted on graphs but in a developmental stage specific manner. This analysis revealed interesting patterns, for instance in a set of 130 DEPs, chromosome 6B contributed to the highest number of DEPs at 4 DPA, chromosome 3A at 8 DPA, and chromosomes 5B and 3B at 12 DPA (Figure S6A). Whereas, in the second set of 306 DEPs chromosomes 2D and 4D of CS and chromosomes 5B and 4D of P271 contributed most DEPs in 4 vs. 8 DPA comparison, and chromosomes 3B and 4D of CS and chromosomes 2B, 4D, and 6D of P271 in 12 vs. 8 DPA comparison (Figure S6B).

**Figure 9:**
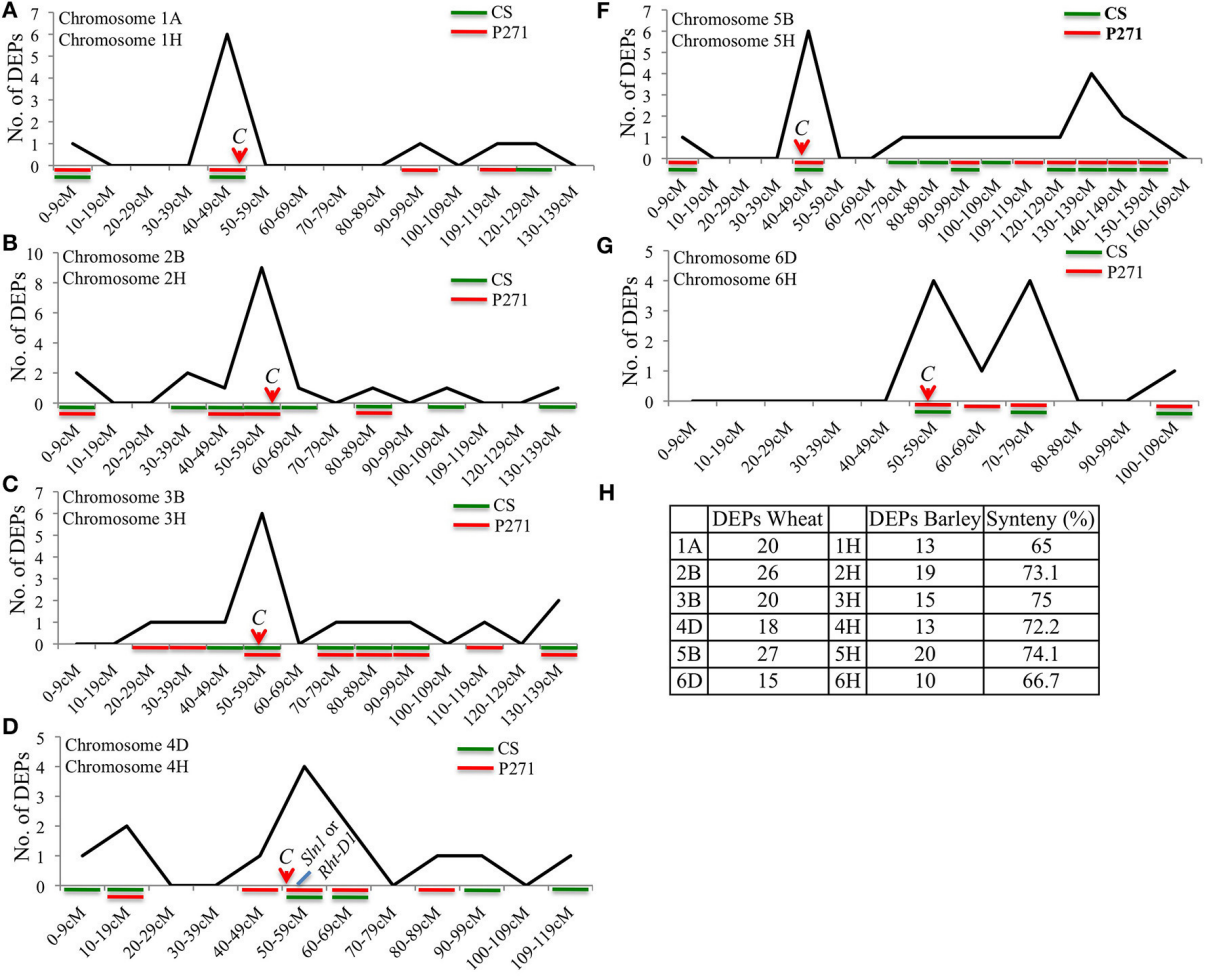
Chromosomal distribution of 306 DEPs shown on syntenous barley chromosomes 1H **(A)**, 2H **(B)** 3H **(C)**, 4H **(D)**, 5H **(F)**, and 6H **(G)**. Genotypes contributing for DEPs were also shown in each figure. Level of synteny between wheat and corresponding barley chromosomes is shown in **(H)**. C = centromere.

Since wheat contigs are not yet anchored genetically we used the barley genomic DNA sequence as reference to determine the distribution of DEPs within the selected chromosomes. Barley and wheat both belong to tribe Triticeae of Gramineae family and were earlier demonstrated to share close synteny with each other. Analysis of the selected chromosomes, which contributed largely to the DEPs, showed a range of 42.9–100% synteny between wheat and barley chromosomes in a set of 130 DEPs (Figure S7), and 65–75% synteny in a set of 306 DEPs (Figure 9). Interestingly, distribution of DEPs on each analyzed chromosome showed a bias toward the centromeric or pericentromeric region of the chromosomes (Figure 9 and Figure S7). Genotypes contributing to the DEPs were also shown in Figure 9, where in all but chromosome 2B/2H DEPs were largely contributed by P271. An approximate location of the barley *Slender1* (*Sln1*) gene, a homolog of the wheat *Rht-D1* gene was also determined. Interestingly, it mapped to barley chromosome 4H in a region coincident with the region populated by several DEPs (Figure 9). This observation might have some implication in the future breeding activities intended to increase grain yield in common wheat.

## Discussion

Considerable amount of research have been carried out on wheat and barley grain development using proteomics approaches (Majoul et al., 2004; Kim et al., 2010; Dong et al., 2012; Ge et al., 2012; Ma et al., 2014; Zhang et al., 2015; Kaspar-Schoenefeld et al., 2016; Yang et al., 2016). However, most of it is focused on mid to late grain developmental stages, especially around the grain-filling phase (Vensel et al., 2005; Dupont, 2008; Dupont et al., 2011; Dong et al., 2012; Ma et al., 2014). In this study, two wheat cultivars P271 and CS were selected to study expression differences during early grain development at protein level. For this purpose protein profiles of two wheat cultivars at three grain developmental stages were investigated using iTRAQ labeling and Q Exactive LC-MS/MS system. Proteomics approach adapted during this study provided deeper insight into the biochemical processes taking placed during the early grain development in common wheat.

We identified more than 3,600 proteins that accumulate during early grain development (from 4 to 12 DPA) in two wheat cultivars. Number of identified proteins suggested that the samples sufficiently covered the grain proteome during the early grain development. In contrast to the former studies number of proteins identified to be involved in the early grain development was high in the preset study, which signifies that a combination of iTRAQ labeling and fractionation of the alkylated and trypsinated peptides prior to LC-MS/MS analysis is far more superior than the previously used methods (cf. Materials and Methods, section High-pH Reversed-Phase Chromatography; Nadaud et al., 2010; Guo et al., 2012). In this study, a total of 130 DEPs were identified between two wheat cultivars and 306 DEPs were identified from the comparisons between different developmental stages in CS and P271 (Figure S3). Genomic distribution of 130 DEPs exhibited a bias for chromosomes 1A, 1B, 2A, 3A, 4B, 4D, and 5D. The analysis suggested that three A-subgenome, two B-subgenome and two D-subgenome chromosomes have contributed to the large grain size in P271 and among these chromosomes 1A, 1B, 2A, and 4D made the largest contributions. Interestingly, in a number of former studies major QTLs for yield and yield contributing traits were identified on these chromosomes (Zhang et al., 2014; Tadesse et al., 2015; Mahjourimajd et al., 2016). Moreover, specifically, major QTLs for grain length (Giura and Saulescu, 1996; Manickavelu et al., 2011; Okamoto et al., 2013; Wu et al., 2015), thousand-grain weight (Jia et al., 2013; Wu et al., 2015), and grain size (Gegas et al., 2010; Simmonds et al., 2014) were mapped to chromosome 4D. A major dwarfing gene *Rht-D1* with a pleiotropic effect on grain weight was also assigned to chromosome 4D.

Previous work on the large grained EMS (ethyl methanesulfonate) induced wheat mutant unraveled that proteins accumulating during early grain development largely represent eight biochemical processes: Stress/defense, carbohydrate metabolism, protein synthesis/assembly/degradation, storage protein biosynthesis, energy production and transportation, photosynthesis, transcription/translation, and signal transduction (Zhang et al., 2015). Similarly, a study conducted in barley on the early grain development emphasized on the accumulation of proteins related to cell cycle regulation, protein synthesis, defense to diseases or oxidative stress, and energy production via photosynthesis (Kaspar-Schoenefeld et al., 2016). These observations are in congruence with our own finding where most of the DEPs fall under the similar functional categories (Figures 3–6).

The central metabolism [glycolysis and tricarboxylic acid cycle (TCA)] provides most of the energy during seed development. The work of Guo et al. (2012) showed that during the early stages of wheat grain development (from 6 to 11 DPA), most of the proteins involved in glycolysis, TCA cycle and/or nitrogen metabolism show down-regulation. It was consistent with our result, where most of the proteins related to TCA cycle and glycolysis were down-regulated in both P271 and CS. Carbohydrate metabolism is an important process which plays an important role during early grain development. It includes energy metabolism-related proteins, in particular the ones involved in glycolysis/gluconeogenesis and TCA (Fernie et al., 2004; Ma et al., 2014; Gu et al., 2015). In the present work we identified specific proteins involved in TCA cycle that showed more suppression in P271 in comparison with CS at three developmental stages. These results imply that the TCA cycle is more active in CS than P271 during early grain development. On the other hand, proteins involved in glycolysis/gluconeogenesis process showed higher expression in P271 in comparison with CS at 4 and 8 DPA. These differentially expressed proteins involved in energy metabolism in different cultivars are likely responsible for the difference in cultivar performances (Guo et al., 2012). GAPDH and glucose-6-phosphate isomerase (GPI) are two key enzymes involved in starch catabolism (Sparla et al., 2002; Guo et al., 2011). The relative abundance of GAPDH was also found to be lower in P271 in comparison with CS.

During early grain development the cell number in endosperm increases rapidly, implicating requirement of amino acids, specific co-factors and vitamins, and cytoskeleton related proteins like actin and tubulin. Inline with the hypothesis tubulin-specific chaperone A (W5G2H3) showed up-regulation during 4–8 DPA only in P271. This observation is in agreement with our hypothesis that large grained P271 requires more cytoskeleton related protein than CS. Energy requirement during the cell differentiation stage is particularly high (Gutierrez-Marcos et al., 2006). Glycolysis, TCA cycle and the mitochondrial electron transport chain are the main process in endosperm cells that feed the energy requirement (Fernie et al., 2004).

14-3-3 proteins function as homo or heterodimers and bind a large number of differentially phosphorylated substrates to regulate a wide array of cell signaling and physiological processes (Nadaud et al., 2010; Lozano-Durán and Robatzek, 2015; Zhang et al., 2015). In this study, we identified two 14-3-3 proteins dubbed G5DFC5 and LOGED8 in both cultivars. These proteins showed 4.03 and 3.04-fold down regulation, respectively at 4 DPA in both cultivars, and showed up to 1.9 and 10-fold up regulation of G5DFC5, respectively in CS and P271 at 8 DPA.

## Conclusion

The present work reports qualitative and quantitative characterization of 347 DEPs during early grain development in two wheat cultivars, P271 and CS, and throws light on the central metabolic changes taking place during early development. Interestingly, a similar number of proteins were identified to accumulate during early grain development in different wheat cultivars, albeit their expression profiles differed between the two genotypes. In comparison with CS considerable number of DEPs that largely represent proteins involved in cell wall biosynthesis, starch and protein biosynthesis and stress mechanisms showed up-regulation in P271. This finding is in consistency with the large grain size and high rate of reserve accumulation observed in P271. Collectively our results have provided insight into the wheat grain proteome during early development, and highlighted the differences in the grain proteomes of wheat cultivars with contrasting grain characteristics.

## Author contributions

XG, SR and SW designed the project and amended the manuscript. MY performed the experiments, analyzed the data and drafted the manuscript. JD, NG, HC and DW collected the data and assisted with data analysis. All of the authors read and approved the manuscript.

### Conflict of interest statement

The authors declare that the research was conducted in the absence of any commercial or financial relationships that could be construed as a potential conflict of interest.

## Supplementary material

Figure S1 Transverse section of developing wheat caryopses from cultivars P271 **(A)** and Chinese Spring (CS) **(B)** at three developmental stages. The lower panels show whole sections and the upper panels show higher magnification images of areas of the central and peripheral endosperm, respectively. Sections are stained with toluidine blue. Bar in the upper panel is 20 μm, and the lower panels is 200 μm. P, pericarp; OI, outer integument; II, inner integument; N, nucellus; NP, nucellar projection; A, aleurone; E, endosperm; SE, starchy endosperm; EC, endosperm cavity; C, cavity; NE, nucellar epidermis; OP, outer pericarp; IP, inner pericarp; N&I, nucellus and integument.

Figure S2 Venn diagram showing the number of proteins identified in P271 and Chinese Spring (CS), 12 proteins were uniquely identified in CS, and 2 in P271, whereas 3,678 proteins were indentified in both P271 and CS.

Figure S3 Venn diagrams showing overlap in the two sets of differentially expressed proteins identified from a comparison between wheat cultivars CS and P271 or different grain developmental stages (4 vs. 8 DPA and 12 vs. 8 DPA) in two wheat cultivars.

Figure S4 Bar diagrams showing genomic distribution of differentially expressed proteins (total 130) in wheat: **(A)** subgenomes, **(B)** homoeologous chromosome groups, and **(C)** specific chromosomes.

Figure S5 Bar diagrams showing genomic distribution of differentially expressed proteins (total 306) in wheat: **(A)** subgenomes, **(B)** homoeologous chromosome groups, and **(C)** specific chromosomes.

Figure S6 Genome wide display of the dynamics of DEP expression in **(A)** CS or P271 at three developmental stages 4 DPA, 8 DPA, and 12 DPA (130 DEPs); **(B)** CS and P271 in CS 4 vs. 8, CS 12 vs. 8, P271 4 vs. 8, and P271 12 vs. 8 comparisons (306 DEPs).

Figure S7 Chromosomal distribution of 130 DEPs shown on syntenous barley chromosomes 1H **(A,B)**, 2H **(C)** 3H **(D)**, 4H **(E,F)**, and 5H **(G)**. Level of synteny between wheat and corresponding barley chromosomes is shown in “H”. C, centromere.

Table S1 Details of the mass tags* used to label the different samples and replicates.

Table S2 Cultivar-specific proteins identified in Chinese Spring or P271.

Table S3 Proteins showing 2-fold expression level differences between P271 and Chinese Spring at 4 days post anthesis (DPA).

Table S4 Proteins showing 2-fold expression level differences between P271 and Chinese Spring at 8 days post anthesis (DPA).

Table S5 Proteins showing 2-fold expression level differences between P271 and Chinese Spring at 12 days post anthesis (DPA).
