# Supplementary figures and images for: Pattern of Protein Expression in Developing Wheat Grains Identified through Proteomic Analysis
Source: Front Plant Sci. 2017 Jun 9;8:962. doi: 10.3389/fpls.2017.00962 (PMC5465268; doi:10.3389/fpls.2017.00962)

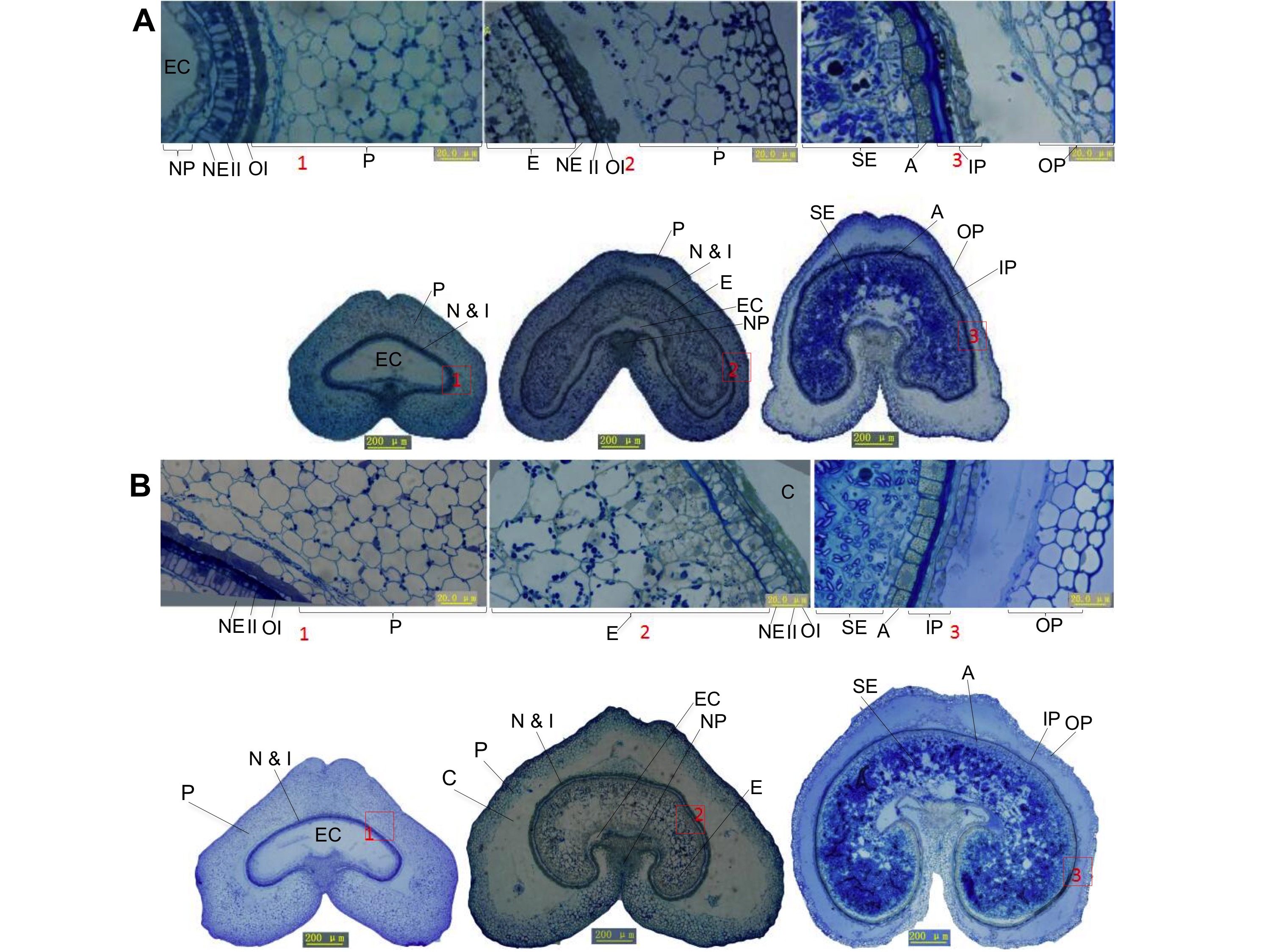

Supplement: Figure S1 — Transverse section of developing wheat caryopses from cultivars P271 (A) and Chinese Spring (CS) (B) at three developmental stages. The lower panels show whole sections and the upper panels show higher magnification images of areas of the central and peripheral endosperm, respectively. Sections are stained with toluidine blue. Bar in the upper panel is 20 μm, and the lower panels is 200 μm. P, pericarp; OI, outer integument; II, inner integument; N, nucellus; NP, nucellar projection; A, aleurone; E, endosperm; SE, starchy endosperm; EC, endosperm cavity; C, cavity; NE, nucellar epidermis; OP, outer pericarp; IP, inner pericarp; N&I, nucellus and integument. [file Image1.JPEG]

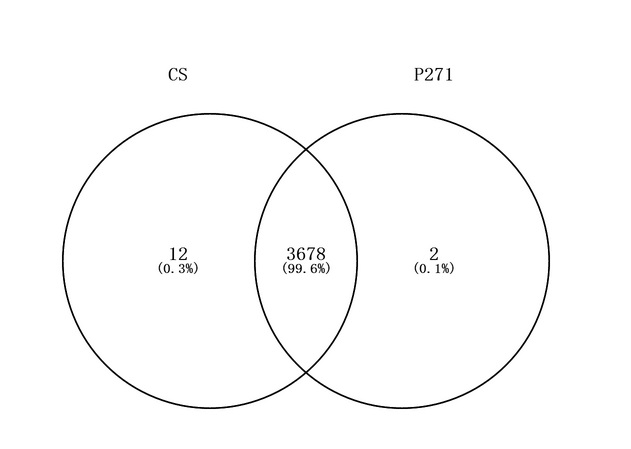

Supplement: Figure S2 — Venn diagram showing the number of proteins identified in P271 and Chinese Spring (CS), 12 proteins were uniquely identified in CS, and 2 in P271, whereas 3,678 proteins were indentified in both P271 and CS. [file Image2.JPEG]

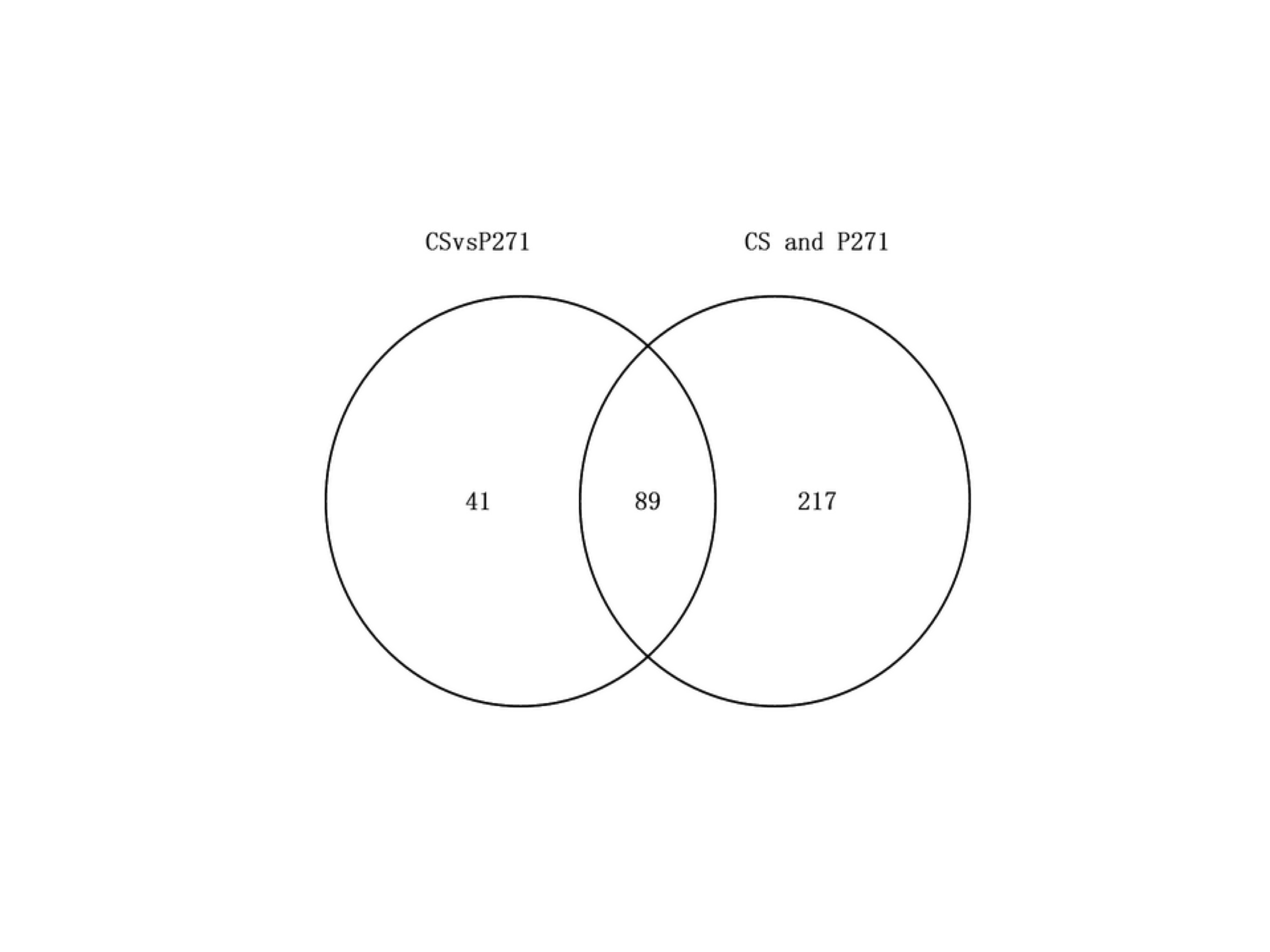

Supplement: Figure S3 — Venn diagrams showing overlap in the two sets of differentially expressed proteins identified from a comparison between wheat cultivars CS and P271 or different grain developmental stages (4 vs. 8 DPA and 12 vs. 8 DPA) in two wheat cultivars. [file Image3.JPEG]

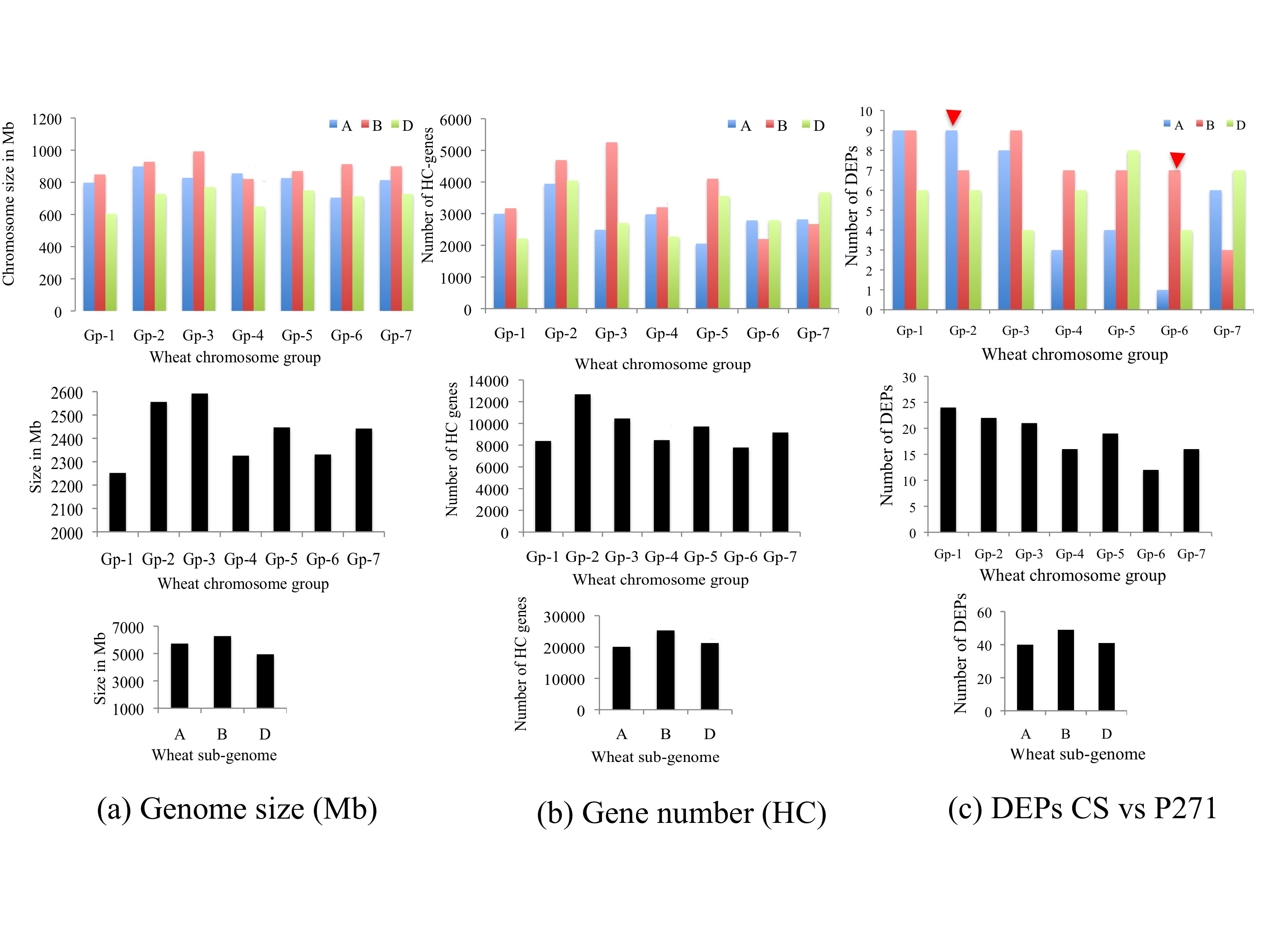

Supplement: Figure S4 — Bar diagrams showing genomic distribution of differentially expressed proteins (total 130) in wheat: (A) subgenomes, (B) homoeologous chromosome groups, and (C) specific chromosomes. [file Image4.JPEG]

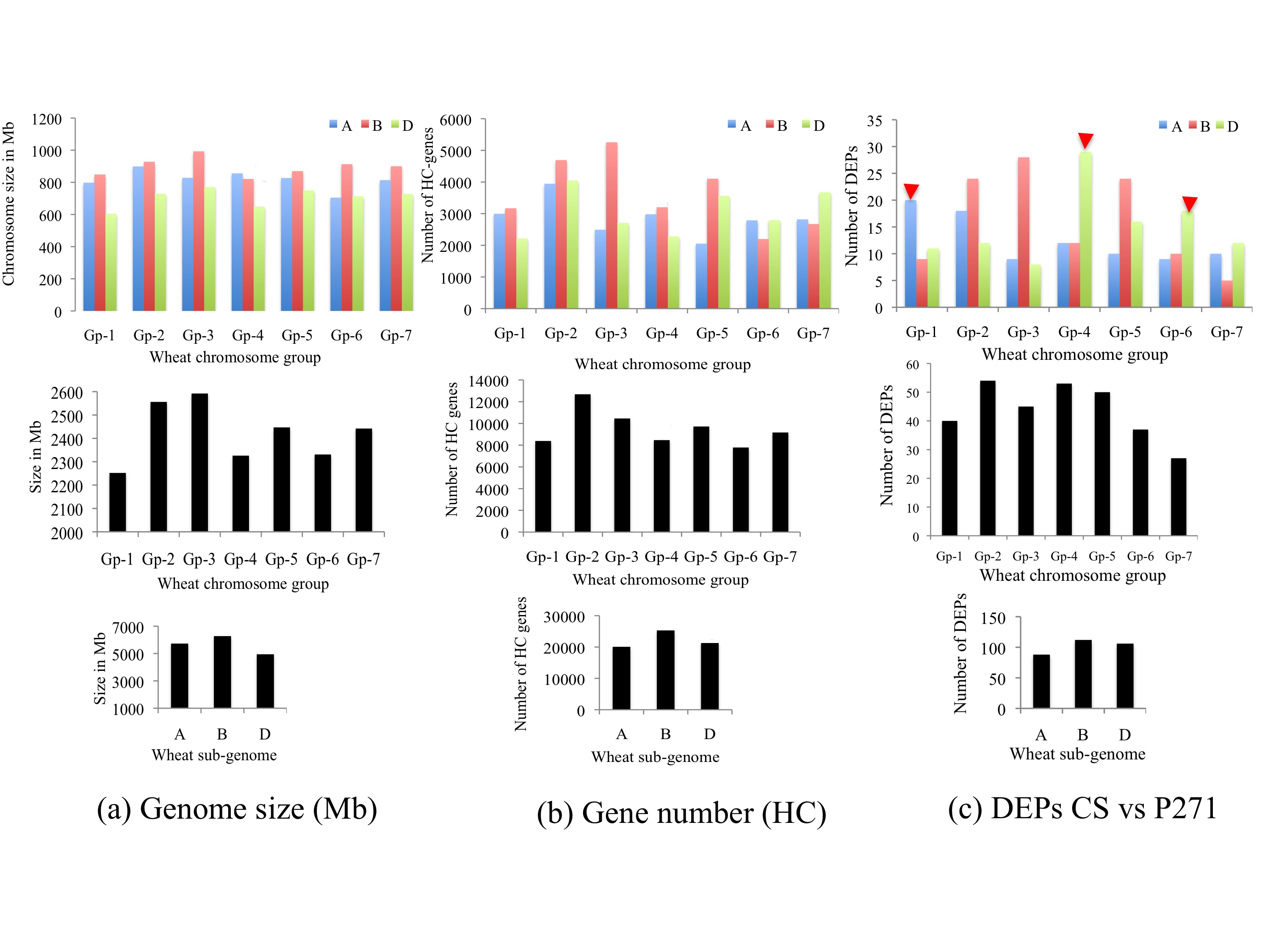

Supplement: Figure S5 — Bar diagrams showing genomic distribution of differentially expressed proteins (total 306) in wheat: (A) subgenomes, (B) homoeologous chromosome groups, and (C) specific chromosomes. [file Image5.JPEG]

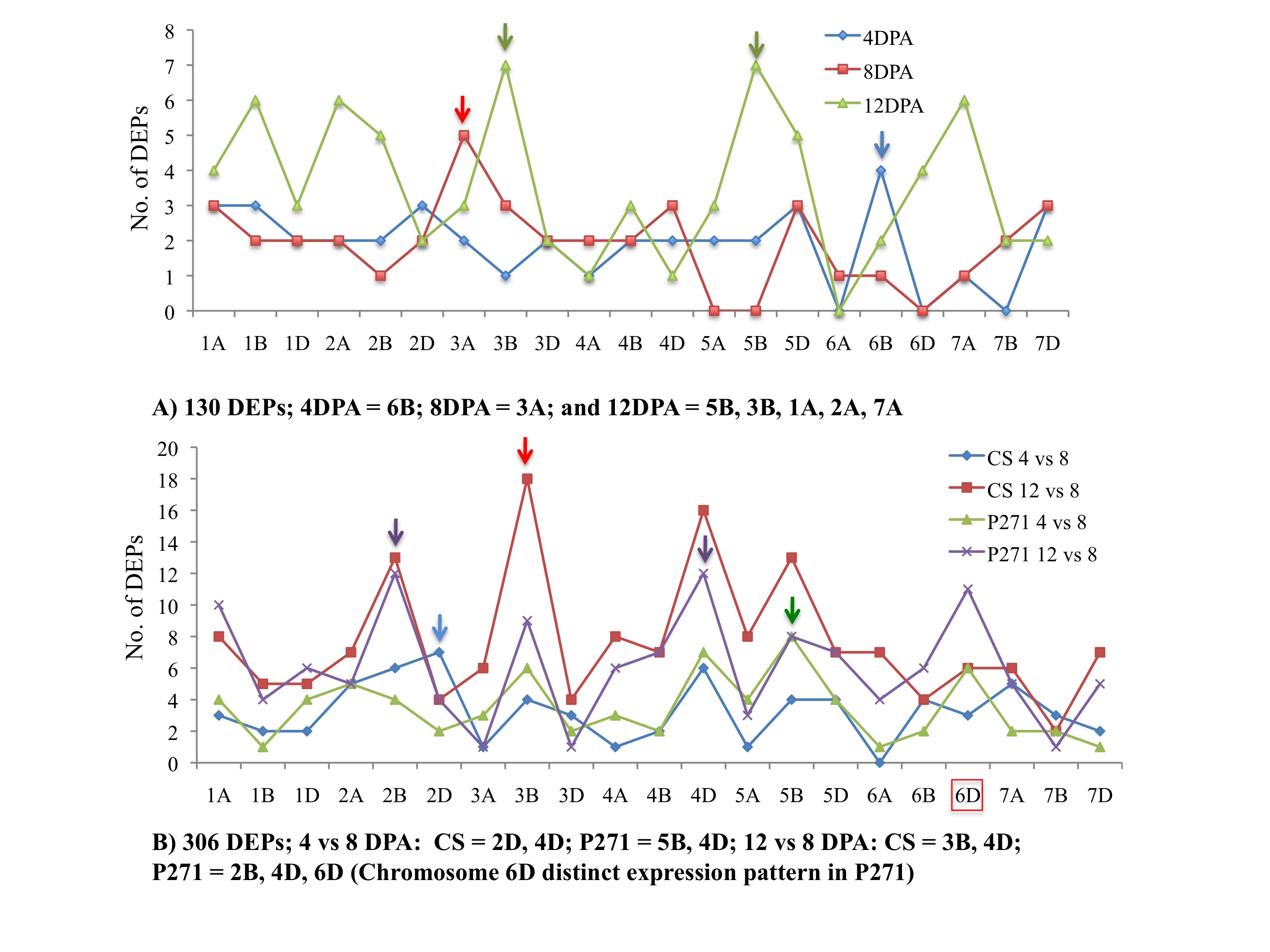

Supplement: Figure S6 — Genome wide display of the dynamics of DEP expression in (A) CS or P271 at three developmental stages 4 DPA, 8 DPA, and 12 DPA (130 DEPs); (B) CS and P271 in CS 4 vs. 8, CS 12 vs. 8, P271 4 vs. 8, and P271 12 vs. 8 comparisons (306 DEPs). [file Image6.JPEG]

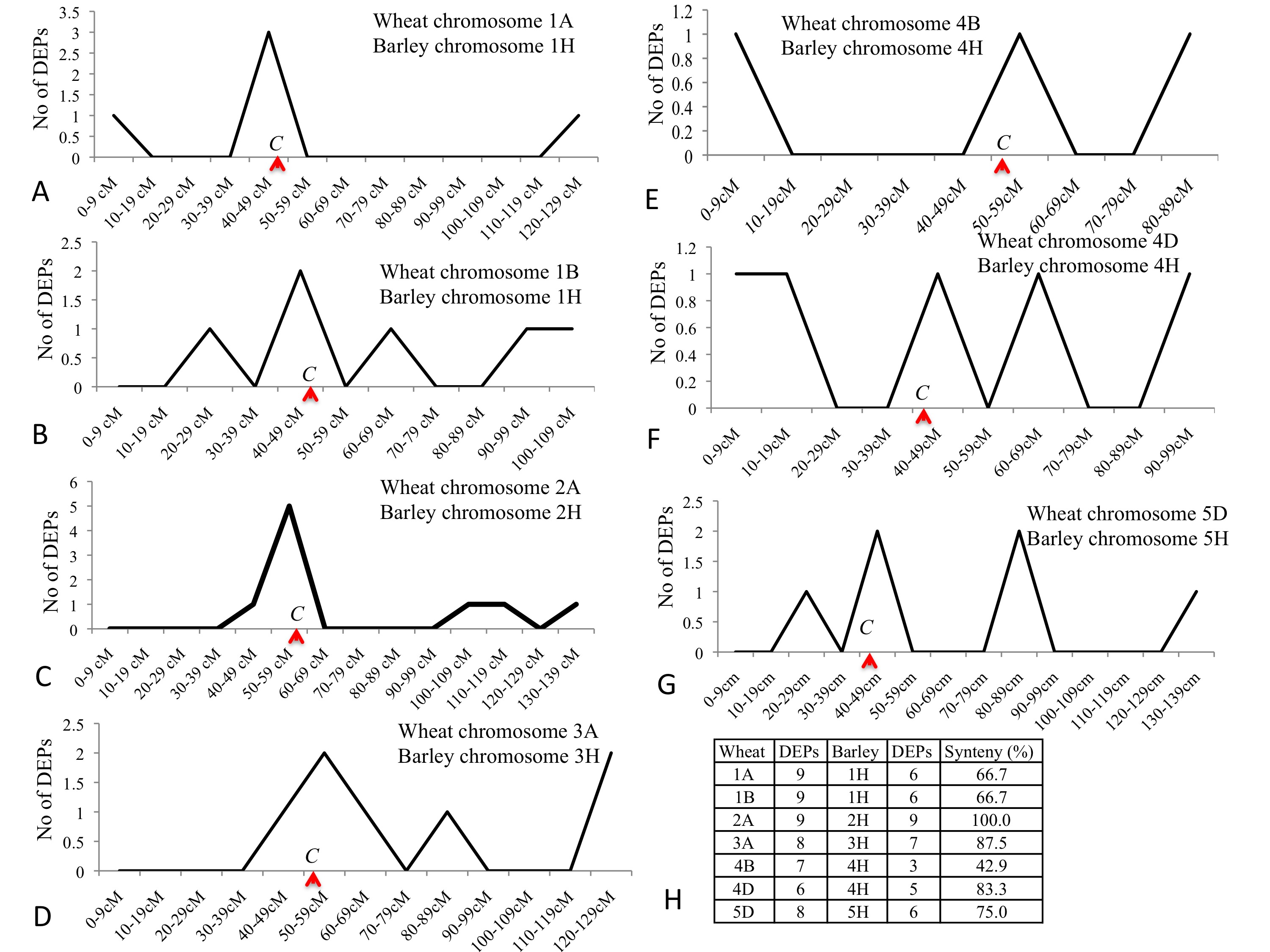

Supplement: Figure S7 — Chromosomal distribution of 130 DEPs shown on syntenous barley chromosomes 1H (A,B), 2H (C) 3H (D), 4H (E,F), and 5H (G). Level of synteny between wheat and corresponding barley chromosomes is shown in “H”. C, centromere. [file Image7.JPEG]
