# Supplementary material for: Pattern of Protein Expression in Developing Wheat Grains Identified through Proteomic Analysis
Source: Front Plant Sci. 2017 Jun 9;8:962. doi: 10.3389/fpls.2017.00962 (PMC5465268; doi:10.3389/fpls.2017.00962)
Supplement: Table S1 — Details of the mass tags* used to label the different samples and replicates. [file Table1.DOCX]

**Table S1.** Details of the mass tags* used to label the different samples and replicates.

| *Genotype* | *Developmental stage* | | | *Replicate* |
| --- | --- | --- | --- | --- |
|  | 4 DPA | 8 DPA | 12 DPA |  |
| Chinese Spring | 113 / 192 | 114 / 191 | 115 / 190 | Bio. replicate # 1 |
| P271 | 116 / 189 | 118 / 187 | 119 / 186 |  |
| Chinese Spring | 113 / 192 | 114 / 191 | 115 / 190 | Bio. replicate # 2 |
| P271 | 116 / 189 | 118 / 187 | 119 / 186 |  |

*Reporter ions / isobaric tag values; DPA = days post anthesis
