# Supplementary material for: Pattern of Protein Expression in Developing Wheat Grains Identified through Proteomic Analysis
Source: Front Plant Sci. 2017 Jun 9;8:962. doi: 10.3389/fpls.2017.00962 (PMC5465268; doi:10.3389/fpls.2017.00962)
Supplement: Table S2 — Cultivar-specific proteins identified in Chinese Spring or P271. [file Table2.DOC]

**Table S2.** Cultivar-specific proteins identified in Chinese Spring or P271

| **Accession #** | **Protein expression** | | **Annotation (GenomeNet)** |
| --- | --- | --- | --- |
|  | **DPA 4 vs 8** | **DPA 12 vs 8** |  |
| **Chinese Spring** | | | |
| W5HZM9 | No | 0.723960087 | Prephenate dehydrogenase |
| W5A2E7 | 1.887991309 | 1.416868508 | acyl-CoA dehydrogenase |
| W5FWD5 | 1.28807503 | 0.742683023 | Subtilase family |
| W5HC20 | 2.190908343 | 0.999218255 | Arsenite-resistance protein 2 |
| W5I5U8 | 1.041531771 | 1.223490179 | Uncharacterized |
| W5E1Y9 | 1.124029756 | 1.15882653 | ATPase family associated with various cellular activities (AAA) |
| W5EEY4 | 1.048239112 | 1.486502945 | Glyceraldehyde 3-phosphate dehydrogenase, NAD binding domain |
| W5GA08 | 0.547292408 | 4.731552899 | Ferredoxin thioredoxin reductase variable alpha chain |
| W5GP97 | 1.467733502 | 1.17193085 | Uncharacterized |
| A0A077RXL7 | 1.030808181 | 1.504154682 | tRNA (guanine(37)-N1)-methyltransferase |
| W5CL64 | 1.096810311 | 0.714867622 | Uncharacterized |
| A0A096UNM7 | 1.397489488 | 1.095470041 | Uncharacterized |
| **P271** | | | |
| W5BAL4 | No | 0.495415986 | Fasciclin domain |
| W5AIG7 | 2.885976613 | 16.17588305 | Proteasome non-ATPase 26S subunit |

DPA = days post anthesis; No = not identified
